# Developing automaticity in neural speech discrimination in typically developing bilingual Italian-German and monolingual German children

**DOI:** 10.1371/journal.pone.0311820

**Published:** 2024-10-23

**Authors:** Theresa Bloder, Tanja Rinker, Valerie Shafer

**Affiliations:** 1 Catholic University Eichstätt-Ingolstadt, Eichstätt, Germany; 2 The Graduate Center, City University of New York, New York, New York, United States of America; Flinders University, AUSTRALIA

## Abstract

Many studies have shown that input in more than one language influences children’s phonemic development. In this study, we examined the neural processes supporting perception of Voice Onset Time (VOT) in bilingual Italian-German children and their monolingual German peers. While German contrasts short-lag and long-lag, Italian contrasts short-lag and voicing lead. We examined whether bilinguals’ phonetic/phonological systems for the two languages develop independently or whether they influence each other, and what role language input plays in the formation of phonetic/phonological categories. Forty five-year-old children (16 monolingual German, 24 bilingual Italian-German) were tested in an oddball design expected to elicit a neural Mismatch Response (MMR). The stimuli were bilabial stop VOT contrasts with the short-lag stop, common to both languages, as the standard. Four deviant VOTs were selected: 92 ms and 36 ms lag for German; 112 ms and 36 ms voicing lead for Italian. Bilingual children’s language background was assessed using a caregiver questionnaire. Italian-German bilingual 5-year-old children and German monolingual controls showed similar MMRs to German long-lag and Italian voicing lead VOT, except for the 36 ms long-lag deviant; this acoustically difficult distinction did not elicit a robust negative MMR in the bilingual children. The lack of a difference between the bilinguals and monolinguals for voicing lead suggests that the amount of input in Italian for the bilinguals was not sufficient to lead to an advantage compared to the monolingual German children. Alternatively, the finding could indicate that voicing lead is easier to discriminate than voicing lag.

## 1 Introduction

Late learning of a second language (L2) can lead to a detectable foreign accent [1] and non-native speech categorization [2], whereas early learners of two languages often develop high proficiency in both languages, with native-like perception skills [3]. While late learning is often defined as beginning after 13 years of age and early learning defined as beginning before 5 years of age, L2 performance may be better modeled as a cline, with less native-like performance with increasing age of onset of L2 learning [4]. This gradual decline in sensitivity towards (foreign) speech sound differences early in life coincides with increased input in the first language (L1). To some extent, this decline with age in the ability to perceive foreign speech sounds in a native-like fashion tracks with the increasing refinement of native speech perception from infancy through grade school for monolingually, as well as bilingually-exposed children. Considerable research indicates that from birth, newborns are equipped with the perceptual prerequisites to acquire any given language [5,6]. During the second half of infants’ first year of life, through language experience, speech perception is modulated so that infants show different behavior to speech sounds from the ambient language compared to speech sounds from non-native languages [7,8]. Specifically, children’s discrimination of non-native speech sound contrasts that are not relevant (i.e., not phonemic) in the native language diminishes. This perceptual change has been called perceptual narrowing or neural commitment [9].

However, development of language skills in a bilingual individual is multifaceted and influenced by a wide array of factors including age of acquisition, but also a range of other factors such as amount of input, amount of language use, context of input/use, quality of input, and sociolinguistic status of a language. Language experience for bilingual children is necessarily split between the heritage and the societal language [10], but with different proportions of the two (or more) languages across different children [11]. This variability in input and use across children is likely to underlie the mixed findings that have been reported for the trajectory of bilingual children’s speech development [12,13]. It remains unclear whether speech perception and its underlying processes for a child with bilingual input converge with those of a monolingually-exposed child or whether they continue to develop on a divergent and unique pathway, because of the different requirements of a bilingual environment.

## 2 Automaticity in speech perception

In his developmental model, Jusczyk claimed that speech perception becomes increasingly automatic over the course of language development [7]. This concept of automaticity in speech perception has been elaborated further by Strange in her Automatic Selective Perception (ASP) model and extended to the context of L1-L2 processing [14]. The model suggests that speech perception is inherently selective, and that this selectivity is shaped by an individual’s linguistic experience, which influences the perceptual weighting of acoustic cues relevant to the native language. The model further posits that speech perception operates automatically. For L1 speakers, this means that the processing of phonetic information occurs rapidly and without conscious effort via selective perceptual routines (SPRs). These SPRs are a product of extensive input and practice with the native language’s phonetic patterns and enable the categorization of continuous acoustic variations into discrete phonemic categories. This categorical perception simplifies the complex auditory input into manageable units, enhancing processing efficiency. However, these well-established and highly over-learned L1 SPRs are often sub-optimal for identifying L2 phonemes. Especially for the late L2 learner, the extraction of the important cues that are necessary to differentiate L2 phonemes (that are not found in the L1) is, thus, hypothesized to require greater attentional resources to overcome the entrenched perceptual biases from the L1. As a result, even proficient L2 users may show poor (automatic) perception of an L2 contrast, particularly with increasing task difficulty, because the L1 SPRs interfere [14]. In the case that the L1 and L2 SPRs match, L2 discrimination will be accurate but if they do not, L2 users will tend to prioritize the familiar acoustic cues of their L1 and assimilate unfamiliar L2 sounds into their closest L1 phonemic category. For example, Japanese adults show poor discrimination of English /l/ vs. /r/, a contrast that is not phonemically relevant in Japanese [15], but good discrimination of English vowels that differ in duration (e.g., /ʌ/ “hut” versus /ɑ/ in “hot”) because the Japanese vowel system distinguishes vowels in terms of length [2]. Shafer extended the ASP model to development and hypothesized that this automaticity of speech perception in the L1 develops over the first four years of life [16]. Still, the question remains how children who grow up simultaneously exposed to two phonetic systems with partially overlapping phonemic inventories establish SPRs and subsequently process the speech sounds in both of their languages.

## 3 Neural indices of automatic speech sound perception

Automaticity of speech perception can be indexed using the electroencephalogram (EEG) in an Event-Related-Potential (ERP) oddball paradigm designed to examine auditory discrimination. This oddball paradigm elicits a discriminative brain response, called the Mismatch Negativity (MMN) [17,18]. The MMN indexes the brain’s pre-attentive detection of an infrequent change in an auditory stimulus after being presented with a series of repeated stimuli. In adults, it is observed as a frontally-distributed negativity peaking between 100 and 250 ms after stimulus onset [19]. It is computed by subtracting the brain’s responses to the repeated stimulus (i.e., the standard) from those of the infrequent stimulus (i.e., the deviant). As neural discrimination between a standard and a deviant becomes more difficult, the MMN shifts later in time and becomes smaller in amplitude [18].

Addressing the topic of developing automaticity, several studies have revealed a discriminative brain response in infants to a deviant in an oddball paradigm. However, in contrast to the adult MMN-pattern, in these studies of infants and young children, both increased positivity and increased negativity to the deviant have been reported, and the latency of the negative response has been found to generally appear later than found for adults [20–22]. For these reasons, the term “Mismatch Response” (MMR) is used. The accumulating research indicates that age and stimulus properties modulate whether a positive MMR (pMMR), a negative MMR (nMMR) or both will be observed, and the amplitude, and latency observed for these responses. The pMMR, that appears to be more prominent in infants, declines with age [23], while the nMMR, which is likely to be equivalent to the MMN observed in adults, is more consistently found in children after four years of age [24–26]. (Note that these studies typically refer to the nMMR as the MMN, which is what we will do from here on as well.) However, the pMMR and MMN can overlap in time, and thus cancel each other out (as in the preschoolers in [23]).

Moreover, there is evidence for a tradeoff between the pMMR and MMN amplitude dependent on the degree of difference, and thus the phonetic difficulty, of a contrast. For instance, Cheng and colleagues revealed a pMMR to a consonant contrast and an MMN to a vowel contrast in 4-to-6-year-old children. The consonant contrast was phonetically more similar than the vowel contrast [27]. Specifically, increased phonetic difficulty may be defined as less phonetic difference between a pair of speech sounds in terms of a target acoustic parameter. For example, the English vowels /ʌ/ as in “hut” versus /ɑ/ as in “hot”, which differ little in first formant (F1) and second formant (F2) frequencies, are more difficult to discriminate than /i/ in “heat” versus /ɑ/ in hot”, which differ widely in F1 and F2 frequencies [2].

In addition, and particularly relevant for the context of bilingual language acquisition, speech perception development is modulated by language experience. Cross-linguistic studies of adults reveal attenuated MMNs to non-native speech sounds when these are non-contrastive in the native language [28]. Adult learners of a second language may continue to show attenuated MMN to L2 speech sounds that are non-contrastive in the first language [2,29].

Especially in children, the interpretation of the MMR pattern is complicated because both maturational, experiential, and attentional factors can all influence whether a pMMR, the MMN or both can be observed. Observation of a pMMR and no MMN may indicate immaturity or lack of experience. Observation of the MMN when attention is drawn away from the stimuli suggests sufficient maturity and experience to support categorizing two speech sounds as different phonemes. However, attention to the stimuli can increase the negativity of the MMN, as seen in studies of adults [30,31]. Infants and young children can succeed in speech perception tasks even before they have established efficient SPRs, because they can use attentional resources to support discrimination [32]. Thus, in children the observation of a consistently-present MMN to a speech contrast (with or without attention), indicates sufficient maturity of auditory cortex, and sufficient linguistic experience to support automatic recovery of the phonetic information needed for lexical access [33].

The prediction from this view of MMRs is that the absence of an MMN to a native-language speech contrast in a young (bilingual) child indicates insufficient experience for discrimination to be automatic. Contrasts that are (acoustic-)phonetically more similar, will require more experience. For children acquiring more than one language, the MMR pattern to speech contrasts in each of the two languages should reflect both the amount of experience and the degree of phonetic similarity. Specifically, the amplitude of the MMR will be directly related to these two factors. However, it is also possible that bilingual experience has a more complex effect. That is, it is possible that children exposed to two languages where the phonological information conflicts, do not fully commit to one of the patterns. In this case, bilinguals may develop a different system that requires attentional resources to select the appropriate phonological system. Otherwise, the bilingual child will need to favor one system over the other [34].

Another change-detection-related ERP response that has been described in children is the late discriminative negativity (LDN), which is visible 300–550 ms after stimulus onset [35]. This ERP component has also been referred to as the late MMN [36]. Less is known about which processes this late negativity indexes or by which conditions it may be influenced. Nevertheless, an observed late MMN is considered evidence of discrimination [37]. It has been suggested that the late MMN reflects the reorientation of attention after a distracting stimulus [38]. However, this hypothesis has inconsistent support from previous research [39]. Still, this late MMN is considered an important indicator for the development of speech processing in toddlers, especially because the MMR in the earlier time frame is often not significant to the subtle speech contrasts that are of particular relevance in studies of language development [40].

Overall, the findings for bilingual children are somewhat mixed in the very few studies that have been undertaken to date. Datta and colleagues found that the MMRs of 8-to-11-year-old bilingual children were indistinguishable from those of their monolingual peers (using an /ɪ/ vs. /ε/ contrast) [39]. Younger children, however, showed a different pattern to this /ɪ/ vs. /ε/ contrast [40,41]. Specifically, bilingually-exposed children under four years of age showed a more positive response than those with monolingual English input [40,41]. This finding was interpreted to indicate that the MMN was emerging earlier for children exclusively exposed to the language of this contrast. In another study, increased input in the language matching the target stimuli (English or Spanish) resulted in a greater negativity of the MMR, and the amount of input in each of the two languages was relevant [42]. The authors suggested that increased negativity (i.e., the amplitude of the MMN) emerged with neural commitment to the native language speech sounds. A few studies have focused specifically on children with bilingual experience between 4 and 8 years of age. Rinker and colleagues examined neural discrimination of a German vowel contrast (/ε/ vs. /e/) that is phonemic in German but not in Turkish in five-year-old bilingual Turkish-German children [43]. They found that, compared to their German monolingual peers, the MMN amplitude in bilinguals was significantly reduced despite their immersion in a German environment for at least two years. However, their brain response to a vowel contrast (/i/ vs. /y/), which exists both in German and in Turkish did not yield significant group differences (i.e., both groups showed an MMN). A different study, however, revealed native-like neural discrimination of a French vowel contrast by Finnish children after only a few months of being immersed in a French language immersion context [44]. The different findings in these studies could be related to several child-external factors (i.e., input quantity and quality, age of starting to acquire the L2). Given the dearth of studies, more research is necessary to understand how these various factors interact.

## 4 The present study

To this end, the current study examines the impact that specific language experience poses on bilingual children’s language development, particularly concerning children’s pre-attentive discrimination of language-specific phonemic cues that phonetically differ between their two languages. More specifically, we tested how bilingual Italian-German children living in Germany discriminate and process voicing contrasts in bilabial stop consonants. We explore how children’s relative amount of current input in their heritage and the societal language influenced neurophysiological correlates of language processing (indexing automatic neural speech sound discrimination). We consider the questions of whether and how it is possible for young children to become automatic in processing two phonetic systems, if the cues for a category between the two languages are in conflict. Bilabial stop consonants that differ in laryngeal properties in initial word/syllable position were selected because the phonetic properties used to distinguish /ba/ from /pa/ differ in the two target languages. Specifically, German contrasts a short-lag Voice Onset Time (VOT) with a long-lag, aspirated VOT, whereas Italian contrasts short-lag VOT with a prevoiced or voicing lead VOT, where laryngeal voicing begins before the release of the consonant (opening of lips). German orthography represents short-lag VOT as “b” and long-lag VOT as “p”, whereas Italian orthography represents prevoiced VOT as “b” and short-lag VOT as “p”. German speakers assimilate Italian prevoiced and short-lag bilabial stop consonants and perceive them as members of the German “b” category, whereas Italian listeners assimilate German short-lag and long-lag aspirated bilabial stop consonants and perceive them as members of Italian “p”. Thus, the two languages are in conflict regarding how to categorize short-lag VOT (phonetically represented in brackets as [p]).

Moreover, we posed the question of whether bilingual children differ from their monolingual peers in the development of SPRs and, thus, their automaticity in speech sound processing. Specifically, we address the following research questions:

**RQ1**: What impact does language experience have on Italian-German bilingual children’s automatic speech sound processing in their two languages?

Hypothesis 1: We predict a robust presence of a negative component of the MMR to a German-like VOT difference (short-lag [pa] versus aspirated [p^h^a]), indicating automaticity in speech processing, in four-to-five-year-old monolingual German children [24]. Considering that bilingual children’s language experience with either one of their two languages is inevitably reduced compared to their monolingual peers, we further predict less automatic neural speech sound discrimination of German-like VOT in the bilingual Italian-German group. This pattern will be indexed by a more positive MMR compared to the German monolingual group [23,42]. Conversely, we hypothesize that bilingual Italian-German compared to German monolingual children will show a more negative MMR to Italian-like VOT deviants because the bilingual children have some experience with the prevoicing found in the Italian voiced sound ([ba]). The monolingual German children are expected to show no MMN to the Italian-like deviant because Italian [ba] and [pa] are assimilated into the same German phoneme category (both perceived as German short-lag [pa]) [3].

Hypothesis 2: In the group of bilingual children, we predict signs of increased (in)voluntary attention to speech sounds, as indexed by overall more negative brain responses when compared to the group of monolingual children [44,45]. This would be in line with previous research suggesting that bilingual speakers more commonly need to rely on details of the surrounding speech stream to identify the target language [46].

**RQ2**: What is the minimum relative amount of language input that allows for monolingual-like automatic speech processing in both languages?

Hypothesis 3: We predict the polarity (negativity vs. positivity) and amplitude of the MMR to be modulated by bilingual children’s Italian versus German experience; specifically, bilinguals who fall below a minimum cutoff of relative amount of language input (i.e., less than 40% for either language [47]) will exhibit more less “committed” signs of neural discrimination (i.e., reduced MMN amplitude/presence of a pMMR and/or later onset) [24].

## 5 Materials and methods

### 5.1 Participants

A total of 40 children with typical language development and normal hearing status between the ages of 3;11 (years;months) and 6;3 participated in this study. Twenty-four of the children were simultaneous or early-sequential bilingual Italian-German speaking children (18 females) with a mean age of 59.4 months (*SD* = 8.5 months) and 16 were monolingual German speaking children (6 females) with a mean age of 61.1 months (*SD* = 6.4 months). The children’s age did not differ significantly across the two groups (*p* = .696). At the time of their participation in this study, all children were living and attending a kindergarten in Germany. All bilingual participants had at least one native Italian-speaking caregiver and were exposed to Italian on a daily basis, although to varying degrees (see Table 1 for an overview of bilingual children’s relative amount of language input and output; for bilingual children’s individual measures of language input and output see S1 Table in S1 File).

**Table 1:** Overview of bilingual Italian-German speaking children’s current language experience as assessed with the Language Background Questionnaire (LBQ). Measures of relative amount of language input and output are displayed in percent (%). Due to one family failing to return the completed questionnaire, *n* = 23 are available.

Relative amount of current language input	Italian	*M* = 43.71, *SD* = 18.33
	German	*M* = 56.29, *SD* = 18.33
Relative amount of current language output	Italian	*M* = 37.03, *SD* = 25.02
	German	*M* = 62.97, *SD* = 25.02

Twenty-two of the bilingual children were born and raised in Germany and two in Italy; those two had moved to Germany before three years of age. Participants with two Italian-speaking caregivers had been exposed to German for a minimum of two years (Table 2 provides an overview of caregivers’ language background). All but one of the bilingual Italian-German children were enrolled in a bilingual Italian-German kindergarten program. Their dual language environment thus provided them with frequent language input from multiple speakers in both Italian and German.

**Table 2:** Caregivers’ language background as assessed by the Language Background Questionnaire (LBQ). **In** the Can-Do-Questionnaire, caregivers additionally had to rate their language proficiency in German and Italian for a variety of different oral and written competences on a scale from 1 to 5 (5 = very good, native-like language skills). Note that a great proportion of caregivers are considered heritage speakers of Italian themselves (58.9% of the mothers and 41.67% of the fathers); meaning that they were born and raised in Germany by Italian speaking parents.

		Mothers (*n* = 23)	Fathers (*n* = 22)
Language(s) spoken	German only	17.39%	31.82%
	Italian only	8.70%	13.64%
	German and Italian*	73.91%	54.55%
	*including Second language learners of German Heritage speakers of Italian Heritage speakers of German Early sequential bilinguals (L1 Italian)	41.18% 58.82%	33.33% 41.67% 8.33% 16.67%
Self-rated language skills	German	*M* = 4.57, *SD* = .87	*M* = 4.12, SD = 1.32
	Italian	*M* = 3.71, *SD* = 1.63	*M* = 3.57, *SD* = 1.86

All monolingual German children had two monolingual German-speaking caregivers, were born, and raised in Germany and were attending a monolingual German kindergarten program and had no experience with Italian (or similar VOT languages, such as Spanish).

The study was approved by the Ethics Committee of the Catholic University Eichstätt-Ingolstadt. Participant recruitment took place between 04.02.2020 and 01.07.2021. Written consent was obtained from all caregivers/children’s legal guardians according to the Declaration of Helsinki.

Monolingual German and bilingual Italian-German children did not differ regarding measures of German language performance (as assessed with a set of morpho-syntactic subtests of the *Linguistische Sprachstandserhebung Deutsch als Zweitsprache* (LiSe-DaZ [48]); see Table 3 for the measured linguistic competences and the respective scores according to group), or regarding a measure of nonverbal intelligence (as assessed with the German adaptation of Raven’s Colored Progressive Matrices (CPM) [49], ps ≥ .126; see Table 3.

**Table 3:** Overview of children’s German language performance assessed with the LiSe-DaZ and nonverbal intelligence assessed with the CPM according to group (monolingual German vs. bilingual Italian-German). For the LiSe-DaZ subtests verb placement and subject-verb-agreement the maximum score is 4; for the subtests word classes and case markings T-scores are displayed.

		Monolingual German (*n* = 16)	Bilingual Italian-German (*n* = 24)
LiSe-DaZ	verb placement	*M* = 4.00, *SD* = .00	*M* = 3.71, *SD* = .46
	subject-verb-agreement	*M* = 3.56, *SD* = .96	*M* = 3.54, *SD* = .98
	word classes	*M* = 51.76, *SD* = 3.71	*M* = 51.84, *SD* = 8.77
	case markings	*M* = 56.81, *SD* = 10.12	*M* = 55.29, *SD* = 14.12
CPM	raw scores	*M* = 16.56, *SD* = 3.43	*M* = 16.17, *SD =* 5.33

### 5.2 Measures of quantity of children’s language input

Children’s current relative language input was measured by means of a caregiver questionnaire to gain an objective estimate of the average proportion of the time participants heard and spoke their heritage (Italian) compared to the societal language (German) during a typical week of their lives. The language background questionnaire (LBQ) was adapted from the two questionnaires used in [50] and, according to the caregivers’ preference, was provided either in Italian or in German.

The main information that was gathered can globally be classified into two categories: (1) language use in the home; that is how much Italian and/or German each family member (e.g., each caregiver, sibling, or any other adult living in the home) spoke to the child (input) and how much Italian and/or German the child spoke to each family member (output); and (2) language use outside the home; that is, how many hours per week the child spent outside of the home (e.g., in kindergarten, with another caretaker outside of the core-family context, on leisure activities, and/or with friends) and how much Italian and/or German the child heard (input) and spoke (output) during these times. Caregivers were asked to use a seven-point scale defined by frequency adverbs and a percentage scale to estimate the proportion of their children’s Italian compared to German experience in different contexts. To preclude the sum of percentages adding up to more than 100% of total language input, the two languages were combined in the same scale (see below).

- Only German (100% German, 0% Italian)
- Predominantly German, hardly any Italian (90% German, 10% Italian)
- Mostly German, sometimes Italian (75% German, 25% Italian)
- The same amount of German and Italian (50% German, 50% Italian)
- Sometimes German, mostly Italian (25% German, 75% Italian)
- Hardly any German, predominantly Italian (10% German, 90% Italian)
- Only Italian (0% German, 100% Italian)

Based on caregivers’ responses, a compound score representing children’s current language experience (one for their language input and output respectively) was calculated similar to the procedure described by [47]. (Refer to the Supplementary Material for the procedure followed in the computation).

### 5.3 Electrophysiological measures

#### 5.3.1 Stimuli

Natural speech stimuli were recorded by a native speaker of Bengali because the language uses both voicing and glottal laryngeal properties (described as the features of spread glottis and voice). These are long-lag aspirated [p^h^a] = [+spread glottis][-voice], short-lag unaspirated [pa] = [-spread glottis,] [-voice] and prevoiced [ba] = [-spread glottis,] [+voice]). We chose this speaker to avoid a bias towards German or Italian and because this allows equally natural-sounding stimuli at both ends of the continuum. Below, we refer to the [p^h^a] versus [pa] discrimination as German-like and the [ba] versus [pa] discrimination as Italian-like.

The speech recordings were obtained in a sound-shielded booth and manipulated in Praat [51] to create a series of stimuli that ranged phonetically from voicing lead [ba], short-lag [pa], and long-lag [p^h^a]; intended to be perceived as /ba/ and /pa/ by Italian and German speakers, but with different boundaries. After editing, the continuum comprised the following VOT values from [ba] via [pa] to [p^h^a]: -112 ms, -96 ms, -87 ms, -72 ms, -54 ms, -46 ms, -36 ms, -20 ms, -10 ms, 0 ms, 5 ms, 11 ms, 16 ms, 25 ms, 36 ms, 56 ms, 76 ms VOT respectively.

Monolingual Italian (*n* = 11) and monolingual German (*n* = 8) adults performed a behavioral ABX task including the aforementioned continuum of stimuli to select the VOT values for the ERP paradigm. The goal was to identify the boundary between /ba/ and /pa/ for each language group and then to select two stimuli from the prevoiced and two from the long-lag part of the continuum, that were categorized differently from short-lag (0 ms VOT). These two stimuli were one close to the boundary (lesser phonetic difference; i.e., “difficult”), and one far from the boundary (greater phonetic difference, i.e., “easy”), and served as the deviants in the ERP paradigm, with the short-lag stimulus [pa] as the standard. For each language-specific VOT contrast (i.e., voicing lead vs. short-lag for Italian; long-lag vs. short-lag for German), adult participants were presented with 5 trials of three consecutive stimuli (stimulus A, stimulus B, and unknown stimulus X) and had to decide whether the stimulus resembled the first (A) or the second (B) target stimulus. A and B were always a clear exemplar of category endpoints of German /ba/ and /pa/ for German speakers and of Italian /ba/ and /pa/ for Italian speakers. This task identified a boundary around 30 ms VOT for the German /ba/ and /pa/ and around -30 ms for the Italian /ba/ and /pa/ (performance around 50%). The stimuli used in the ERP pattern as the “Easy” deviant contrast was consistently judged as belonging to the target VOT category above 90% for the native listeners.

**Fig 1:**
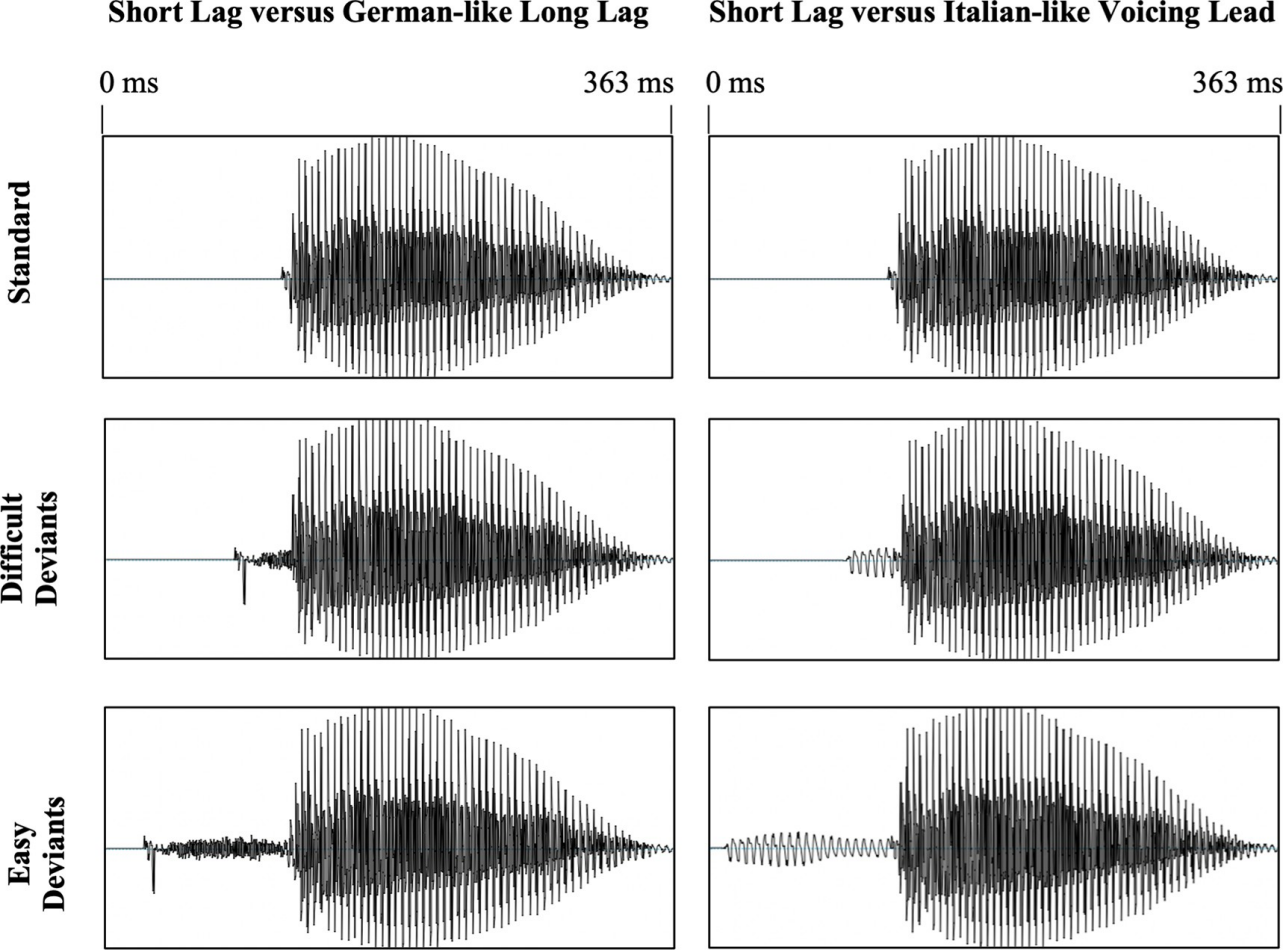
VOT stimuli waveforms. The waveforms of the standard (Short-Lag) and deviant stimuli (German-like Long-Lag vs. Italian-like Voicing Lead; Easy vs. Difficult) used in the EEG experiment. The same standard was used in the two paradigms. The duration of the syllable from stimulus onset to vowel end was 247.66 ms for the Short-Lag standard, 335.93 ms for the Long-Lag Easy 92 ms VOT deviant, 275.99 ms for Long-Lag Difficult 36 ms VOT deviant, 351.19 ms for Voicing Lead Easy -112 ms VOT deviant, and 274.74 ms for Voicing Lead Difficult -36 ms VOT deviant.

The 92 ms VOT stimulus was identified as long-lag on 96.9% of the trials by the monolingual German adults and on 90.9% of the trials by the monolingual Italian adults; the -112 ms VOT stimulus was identified as voicing lead on 81.2% of the trials by the monolingual German adults and on 93.2% of the trials by the monolingual Italian adults. The deviants for the Difficult EEG paradigm were selected such that monolingual adult speakers were able to successfully perceive the stimulus as the target (> 70% correct trials) if it was their native contrast, whereas the success rate in judging the stimulus as one or the other category was at chance level for the non-native distinction: The 36 ms VOT stimulus was identified as long-lag on 71.9% of trials by the monolingual German adults and on 47.7% of the trials by monolingual Italian adults; the -36 ms VOT stimulus was identified as voicing lead on 50.0% of the trials by the monolingual German adults and 77.3% of the trials by the monolingual Italian adults. Fig 1 shows a spectrogram of the stimuli (and the onsets).

#### 5.3.2 Design

The four selected deviant stimuli (one considered”easy” and one considered “difficult” for each language) were used to create two EEG double-deviant oddball paradigms. The Easy paradigm included the 92 ms VOT and the -112 ms VOT deviants. The Difficult paradigm included the 36 ms VOT and the -36 ms VOT deviants. The double-oddball paradigm allowed examination of two deviant stimuli under the exact same conditions so that fatigue or other external conditions would not account for differences in children’s brain responses to the two deviants [52]. Both Easy (i.e., more salient) and Difficult conditions were included because these would provide a more nuanced view of the development of neural discrimination than selecting just one VOT.

Eighty percent of all stimuli were the repeated [pa] standards. The deviants [p^h^a] and [ba] were equally distributed (10% each) among the remaining 20% of tokens. Stimuli were presented in a pseudo-randomized order to allow for at least three consecutive standard stimuli between the presentation of a deviant. The stimuli were presented so that they were be perceived as aligned according to the vowel onset rather than the onset of acoustic information (i.e., prevoicing or aspiration) with the goal to present them with a sense of regular rhythm. The inter-stimulus interval was 600 ms from the offset of the vowel to 122 ms before the onset of the next vowel. As a result, the ISI between vowel offset and burst speech onset differed from each stimulus type (with the longer ISI for the standard [pa]). At the end of the oddball paradigm, each deviant sound was repeated 100 times for use as a control-deviant (also referred to as the deviant’s identity) to which the deviant response was compared for the subsequent analyses [42].

#### 5.3.3 EEG acquisition

The EEG signal was recorded at a 500 Hz sampling rate using a BrainProducts Inc. EEG system via a PC laptop running BrainVision Recorder software. Online bandpass filtering was DC to 131 Hz, and FCz served as the reference. The system includes the LiveAmp 32 amplifier to record the continuous EEG from the scalp using 32 actiCAP slim electrodes mounted in the actiCAP snap electrode cap. Electrode placement included standard placements in the 10/10 montage. Electrodes were filled with SuperVisc electrolyte gel to reduce impedances below 50 kΩ. Active circuits for impedance conversion are directly integrated in the actiCAP slim electrodes. Impedance conversion at the electrode level makes it possible to achieve high signal quality with higher impedances.

#### 5.3.4 EEG preprocessing

The continuous EEG data were processed offline using BrainVision Analyzer software v2.1 (BrainProducts Inc.). After visual inspection of the raw data for each participant, channels contaminated by noise were reconstructed using triangulation and linear interpolation. The signal was re-referenced to the average mastoid reference. An IIR filter (low cut-off: 0.10; high cut-off: 30 Hz) was applied to the signal, followed by a 50 Hz notch filter. Independent Component Analysis (ICA) was used to perform ocular correction. The frontal electrodes (FP1 and FP2) served as a blink marker channels for vertical activity. The difference between FT9 and FT10 electrodes served as a marker for horizontal activity. The procedure was conducted in semi-automatic mode. For each participant, ICA components were inspected visually with respect to their topographic location and relative impact on the data. The components that were contributing to blinks were set to zero. Next, the data was segmented into epochs with interval durations of 200 ms pre- and 900 ms post-stimulus onset. Then artifact rejection was carried out with the criterion of no voltage step of more than 100 μV in the segment. Baseline correction was performed using the 200 ms pre-stimulus amplitude. For each stimulus type (Standard, Voicing Lead deviant, and Long-Lag deviant), segments (-200 to 900 ms) were averaged separately. To ensure that the ERPs to the standard did not include any change-related aspects, post-deviant standards were not included in the standard averages. For each deviant, all children had more than 80 trials; 90% had more than 90 trials that were included in the averages. The mean number of trials for the standard and deviant ERPs did not differ significantly between the two groups (all *p*_*s*_ > .05; see Table 4). The identity MMR (iMMR) was generated by subtracting the averaged brain response the control stimulus from the averaged brain response to the deviant of the same stimulus.

**Table 4:** Overview of the number of trials for each stimulus according to group (monolinguals vs. bilinguals) and paradigm (Easy vs. Difficult).

		Monolinguals	Bilinguals
Short-Lag Standard	Difficult	*M* = 572.55, *SD* = 24.52	*M* = 572.98, *SD* = 20.24
	Easy	*M* = 574.11, *SD* = 18.84	*M* = 575.81, *SD* = 13.57
Voicing Lead Deviant	Difficult	*M* = 95.33, *SD* = 4.27	*M* = 95.58, *SD* = 3.34
	Easy	*M* = 94.11, *SD* = 2.65	*M* = 94.02, *SD* = 2.54
Long-Lag Deviant	Difficult	*M* = 95.16, *SD* = 4.33	*M* = 95.34, *SD* = 3.34
	Easy	*M* = 97.56, *SD* = 3.87	*M* = 97.82, *SD* = 2.54
Voicing Lead Identity	Difficult	*M* = 94.19, *SD* = 5.38	*M* = 94.56, *SD* = 4.92
	Easy	*M* = 95.07, *SD* = 3.99	*M* = 95.56, *SD* = 3.63
Long-Lag Identity	Difficult	*M* = 95.38, *SD* = 4.55	*M* = 94.80, *SD* = 4.67
	Easy	*M* = 95.36, *SD* = 3.25	*M* = 96.29, *SD* = 3.14

#### 5.3.5 EEG analysis

The first step in the analysis was to reduce the ERP data from 32 sites to a model representing the MMR. We used a principal component analysis (PCA) to examine the topography of children’s iMMRs and to determine the optimal electrodes to include in the subsequent analysis. The PCA identified similar topography for component 1 (that accounted for the most variance), which included sites F3, Fz, and F4. These sites have also been reported to show the largest amplitude MMRs in previous studies [40]. To reduce noise unrelated to the stimulus, we averaged across the ERPs recorded at F3, Fz, and F4 for each participant to create the MMR measure used in statistical analyses.

### 5.4 Procedure

Each child was tested individually in a quiet room in one of the kindergartens where they were recruited or at the university. Three monolingual German participants’ behavioral language skills were assessed in their home and in the case of one monolingual and one bilingual child, the entire testing protocol (behavioral tests and EEG) was carried out in a quiet room in their home due to Covid-19 contact restrictions. Data collection extended across 2 to 3 testing sessions (45 to 60 minutes each) and took place on 2 to 3 consecutive days. Due to Covid-19, data collection had to be interrupted abruptly for several months during the spring of 2020, and thus for 6 children there was a wider gap (of about 3–4 months) between the collection of their EEG data and their behavioral measures. Bilingual children’s caregivers completed the LBQ and Can-Do-Questionnaire prior to their participation. To confirm normal hearing status, all children had to pass a hearing screening at 500 Hz, 1000 Hz, 2000 Hz and 4000 Hz (pure tone threshold, 25 dB HL) immediately preceding the EEG recording. To elicit the MMR, during a passive listening task, children were presented with a train of the standard stimulus [pa], occasionally interrupted by either one of the two deviants [ba] and [p^h^a]. No active task was required from the children; they were allowed to watch a muted cartoon on an Ipad screen, while the auditory stimuli were presented binaurally through headphones at 60 dB SPL, delivered via Eprime software (Psychology Software Tools, Pittsburgh, PA, United States). Participants were randomly presented with either the Easy paradigm or the Difficult paradigm first. Due to fatigue and non-compliance, not all children managed to complete both EEG paradigms. Out of the 24 bilingual Italian-German participants, two did not complete the Easy paradigm and another two did not complete the Difficult paradigm. Note that these are not the same children. Similarly, one monolingual German child did not complete the Difficult paradigm.

### 5.5 Data analysis

Based on visual inspection of children’s iMMRs, two time windows of interest were selected. These roughly matched what had previously been reported in the literature. Time window 1 (henceforth the early iMMR) between 120–280 ms (cf. 140–260 ms in [24] for monolingual English-speaking four-to-five-year-olds) and time window 2 (henceforth the late iMMR) between 360–520 ms (cf. 401–490 ms in [37], although for a group of older children). The early versus late MMR are thought to reflect different underlying neural processes and stages of auditory processing [40,45].

To determine whether there was a difference in averaged ERPs to the deviants and their respective identity-control stimulus, for each deviant (Easy Long-Lag, Difficult Long-Lag, Easy Voicing Lead, and Difficult Voicing Lead), a three-way ANOVA with stimulus type (identity ERP amplitude vs. deviant ERP amplitude) and time (divided into four successive 40-ms time intervals: time window 1 vs. time window 2 vs. time window 3 vs. time window 4) as the within-subject measures, and group (bilingual children vs. monolingual children) as the between-subject variable was performed. This procedure was applied to both the early iMMR and the late iMMR target time window. To identify when the early iMMR began, we tested four successive 40-ms time intervals between 120 and 280 ms (120–160 ms, 160–200 ms, 200-240ms, and 240–280 ms). Likewise, we tested four 40 ms time intervals between 360 and 520 ms (360–400 ms, 400–440 ms, 440–480 ms, and 480–520 ms) to determine the onset of the late iMMR. To examine whether there was an effect of stimulus difficulty on the MMR, a three-way ANOVA with difficulty level (Easy deviant vs. Difficult deviant) and target language (Italian-like Voicing Lead vs. German-like Long-Lag) as the within-subject measures and group (bilingual children vs. monolingual children) as the between-subject variable was conducted. This analysis was performed only if there was a significant MMR found for at least one of the stimulus types.

Pearson’s r correlations (for children’s relative amount of language input in %) were run to determine whether there was a relationship between children’s language experience and the mean amplitude of their iMMR.

No Bonferroni correction was applied when the analysis was based on a priori hypotheses, nor when it involved a set of mutually-correlated variables. In all other cases, Bonferroni correction and the value of alpha are specified in the results section.

## 6. Results

**Fig 2:**
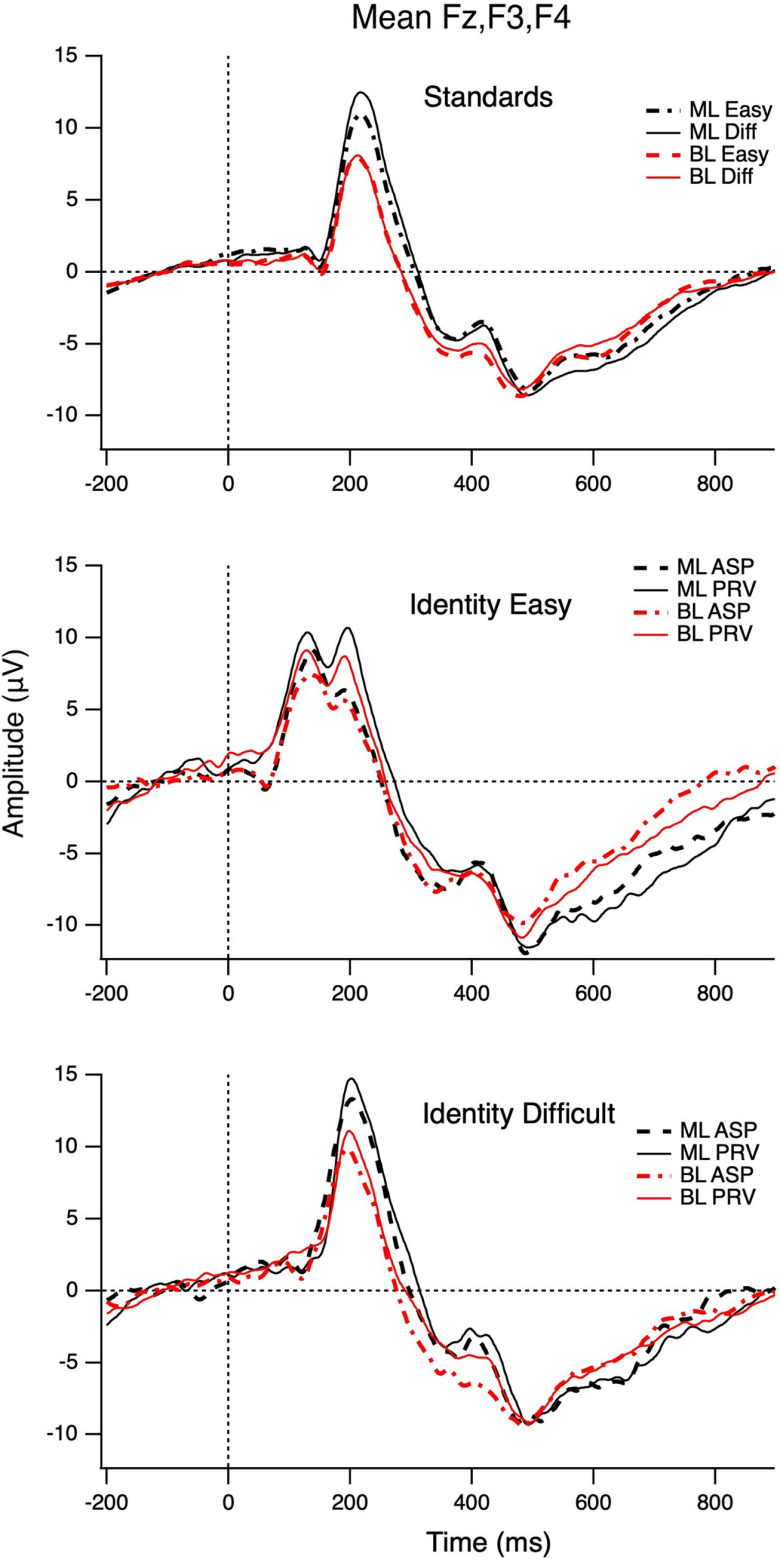
Participants’ auditory evoked potentials. Monolingual (ML) and bilingual (BL) children’s ERPs averaged across frontal sites (Fz, F3, and F4) to the Standard (0 ms VOT) stimulus plotted separately for Easy and the Difficult EEG paradigm as well as to all deviant stimuli (Long-Lag (i.e., aspirated; ASP) Easy and Difficult, Voicing Lead (i.e., prevoiced; PRV) Easy and Difficult) presented in the identity condition.

All children showed typical ERPs to all VOT stimuli, consisting of a large initial positivity (P100) followed by a negativity (N250) [24]; for instance, see Fig 2 for monolinguals’ versus bilinguals’ ERP to the Short-Lag (0 ms VOT) standard stimulus and all deviant stimuli presented in the identity condition. An independent samples *t*-test revealed a significant difference in children’s mean P100 amplitude (*t*(38) = 4.456, *p* < .001). On average bilingual participants (*n* = 24) showed a more negative P100 response (*M* = 3.38, *SD* = 1.90) than their monolingual peers (*n* = 16, *M* = 6.16, *SD* = 1.98). Examination of monolinguals’ versus bilinguals’ mean ERP amplitudes to the different stimuli in MMR relevant time widows (see S2 and S3 Tables in S1 File) further shows that in general bilingual children’s brain responses were more negative than those of their monolingual peers.

For the mixed three-way ANOVAs comparing the ERPs of the deviants to their respective identity stimuli, outliers of 3 *SDs* above/below the mean were excluded from the analyses. The assumption of normality was assessed using Shapiro-Wilks test. As *p*_*s*_ > .05, the null hypothesis that the data were normally distributed was accepted. Levene’s test confirmed homogeneity of variances (*p*_*s*_ > .05). The assumption of covariance of matrices was also met. Only the assumption of sphericity for variables with more than two levels (i.e., time) was not met. Thus, Greenhouse-Geisser correction was used where applicable.

### 6.1 Early iMMR

An overview of children’s mean ERP amplitudes to the various stimuli can be found in S2 Table in S1 File. For the Difficult Long-Lag 36 ms VOT deviant, there was no significant effect of stimulus (deviant vs. identity; *p* = .731) but a significant effect for time (120–160 ms vs. 160–200 ms vs. 200–240 ms vs. 240–280 ms; *F*(1.65,57.85) = 82.152, *p* < .001, η_p_^2^ = .701). Additionally, there was a significant effect of group (monolinguals vs. bilinguals; *F*(1,35) = 5.184, *p* = .029, η_p_^2^ = .129). Generally, independent of stimulus, bilinguals showed more negative ERPs than their monolingual peers across all time points (bilinguals *M* = 5.48, *SE* = .64; monolinguals *M* = 7.78, *SE* = .78). Furthermore, there was a significant interaction effect of time x group, *F*(1.65,57.85) = 4.921, *p* = .015, η_p_^2^ = .123. The results of a post-hoc independent samples *t*-test showed that the difference between monolinguals and bilinguals was non-significant at time point 1 (120–160 ms; *p* = .859) and time point 2 (160–200 ms; *p* = .073) but significant at time point 3 (200–240 ms; *t*(35) = 2.877, *p* = .007) as well as at time point 4 (240–280 ms; *t*(35) = 2.156, *p* = .038). The monolinguals showed a more positive response than the bilinguals (time point 3: monolinguals *M* = 11.68, *SE* = 1.03; bilinguals *M* = 7.83, *SE* = .85; time point 4 monolinguals *M* = 6.03, *SE* = 1.15; bilinguals *M* = 2.82, *SE* = .95).

For the Easy Long-Lag 92 ms VOT deviant, there was no significant effect of stimulus (deviant vs. identity; *p* = .083) but a significant effect for time (120–160 ms vs. 160–200 ms vs. 200–240 ms vs. 240–280 ms; *F*(1.78,62.58) = 109.127, *p* < .001, η_p_^2^ = .757). There was no significant effect of group (monolinguals vs. bilinguals; *p* = .293). No significant interactions between the three factors were found (*p*_*s*_ > .156).

For the Difficult Voicing Lead -36 ms VOT deviant, there was a marginally significant effect of stimulus (deviant vs. identity; *F*(1,34) = 4.033, *p* = .053, η_p_^2^ = .106). All children showed more negative ERPs to the deviant (*M* = 6.06, *SE* = .49) than to its identity (*M* = 7.05, *SE* = .49). Further, there was a significant effect for time (120–160 ms vs. 160–200 ms vs. 200–240 ms vs. 240–280 ms; *F*(1.75,59.32) = 120.333, *p* < .001, η_p_^2^ = .780). Additionally, there was a significant effect of group (monolinguals vs. bilinguals; *F*(1,34) = 11.274, *p* = .002, η_p_^2^ = .249). Generally, independent of stimulus, bilinguals showed more negative ERPs than their monolingual peers across all time points (bilinguals *M* = 5.12, *SE* = .54; monolinguals *M* = 8.01, *SE* = .67). Finally, there was a significant interaction effect for time x group (*F*(1.75,59.32) = 11.521, *p* < .001, η_p_^2^ = .253). The results of a post-hoc independent samples *t*-test showed that the difference between monolinguals and bilinguals was non-significant at time point 1 (120–160 ms; *p* = .823) but significant at time point 2 (160–200 ms; *t*(34) = 2.044, *p* = .049; monolinguals *M* = 9.37, *SE* = .84, bilinguals *M* = 7.20, *SE* = .65), time point 3 (200–240 ms; *t*(34) = 4.192, *p* < .001; monolinguals *M* = 13.36, *SE* = .65; bilinguals *M* = 8.53, *SE* = .82) as well as at time point 4 (240–280 ms; *t*(34) = 4.098, *p* < .001; monolinguals *M* = 7.28, *SE* = .66; bilinguals *M* = 2.55, *SE* = .82); bilinguals showed significantly more negative ERPs than their monolingual peers independent of stimulus (deviant vs. identity).

For the Easy Voicing Lead -112 ms VOT deviant, there was no significant effect of stimulus (deviant vs. identity; *p* = .179) but a significant effect for time (120–160 ms vs. 160–200 ms vs. 200–240 ms vs. 240–280 ms; *F*(1.53,54.90) = 82.152, *p* < .001, η_p_^2^ = .692). There was no significant effect of group (monolinguals vs. bilinguals; *p* = .189). Neither were there any significant interactions between the three factors (*p*_*s*_ > .301).

### 6.2 Late iMMR

An overview of children’s mean ERP amplitudes to the various stimuli can be found in S3 Table in File. For the Difficult Long-Lag 36 ms VOT deviant, there was a significant effect of stimulus (deviant vs. identity; *F*(1,35) = 14.491, *p* = .001, η_p_^2^ = .293), and a significant effect for time (360–400 ms vs. 400–440 ms vs. 440–480 ms vs. 480–520 ms; *F*(1.59,55.47) = 43.958, *p* < .001, η_p_^2^ = .557) but no significant effect of group (monolinguals vs. bilinguals; p = .858). Finally, there was a significant interaction effect of stimulus x group, *F*(1,35) = 7.021, *p* = .012, η_p_^2^ = .167 (monolinguals: deviant *M* = -10.34, *SE* = 1.49, identity *M* = -6.23, *SE* = 1.37; bilinguals: deviant *M* = -8.334, *SE* = 1.23, identity *M* = -7.59, *SE* = 1.13). The results of a post-hoc paired samples *t*-test showed that the difference between children’s ERP amplitude to the deviant and its identity was significant for monolinguals but not for the group of bilinguals (monolinguals: *t*(14) = -4.242, *p* = .001; bilinguals: *t*(21) = -.901, *p* = .378).

For the Easy Long-Lag 92 ms VOT deviant, there was a significant effect of stimulus (deviant vs. identity; *F*(1,35) = 25.026, *p* < .001, η_p_^2^ = .417) and a significant effect of time (120–160 ms vs. 160–200 ms vs. 200–240 ms vs. 240–280 ms; *F*(1.58,55.39) = 48.770, *p* < .001, η_p_^2^ = .582) but no significant effect of group (monolinguals vs. bilinguals; *p* = .492). Furthermore, there was a significant interaction effect of time x group, *F*(1.58,55.39) = 3.547, *p* = .046, η_p_^2^ = .092. The results of a post-hoc independent samples *t*-test showed that the difference between monolinguals and bilinguals was non-significant at any of the four time windows (*p*_*s*_ > .262).

For the Difficult Voicing Lead -36 ms VOT deviant, there were significant effects of stimulus (deviant vs. identity; *F*(1,34) = 4.872, *p* = .034, η_p_^2^ = .125) and time (120–160 ms vs. 160–200 ms vs. 200–240 ms vs. 240–280 ms; *F*(1.53, 52.14) = 49.245, *p* < .001, η_p_^2^ = .601), but not for group (monolinguals vs. bilinguals, *p* = .592). Furthermore, there were no significant interaction effects for any of the factors (*p*_*s*_ > .225). The interaction between stimulus x time approached significance (*p* = .074). A paired samples *t*-test with Bonferroni correction applied (new alpha-level set at .0125) revealed that the difference between the Difficult Voicing Lead deviant and its identity was significant at time point 2 (400–440 ms; *t*(35) = -2.041, *p* = .006; deviant *M* = -6.38, *SE* = .92; identity *M* = -4.10, *SE* = .85), and time point 3 (440–480 ms; *t*(35) = -2.769, *p* = .009; deviant *M* = -9.09, *SE* = .92; identity *M* = -6.86, *SE* = .87), but not at time point 1 (360–400 ms; *p* = .090) and time point 4 (480–520 ms; *p* = .174).

For the Easy Voicing Lead -112 ms VOT deviant, there was a significant effect of stimulus (deviant vs. identity; *F*(1,33) = 15.957, *p* < .001, η_p_^2^ = .326) and a significant effect of time (120–160 ms vs. 160–200 ms vs. 200–240 ms vs. 240–280 ms; *F*(2.25,74.39) = 58.499, *p* < .001, η_p_^2^ = .63.9). There was no significant effect of group (monolinguals vs. bilinguals; *p* = .940). Furthermore, there was a significant interaction effect of stimulus x time, *F*(1.69,55.62) = 4.751, *p* = .017, η_p_^2^ = .126. A paired samples *t*-test with Bonferroni correction applied (new alpha-level set at .0125) revealed that the difference between the deviant and its identity was significant at time point 1 (360–400 ms; *t*(37) = -2.626, *p* = .0125; deviant *M* = -8.23, *SE* = 1.03; identity *M* = -6.32, *SE* = .99), time point 2 (400–440 ms; *t*(37) = -3.127, *p* = .003; deviant *M* = -8.58, *SE* = 1.02; identity *M* = -4.19, *SE* = .85), and time point 3 (440–480 ms; *t*(37) = -3.405, *p* = .002; deviant *M* = -11.48, *SE* = 1.05; identity *M* = -9.67, *SE* = .98), but not at time point 4 (480–520 ms; *p* = .026).

**Fig 3:**
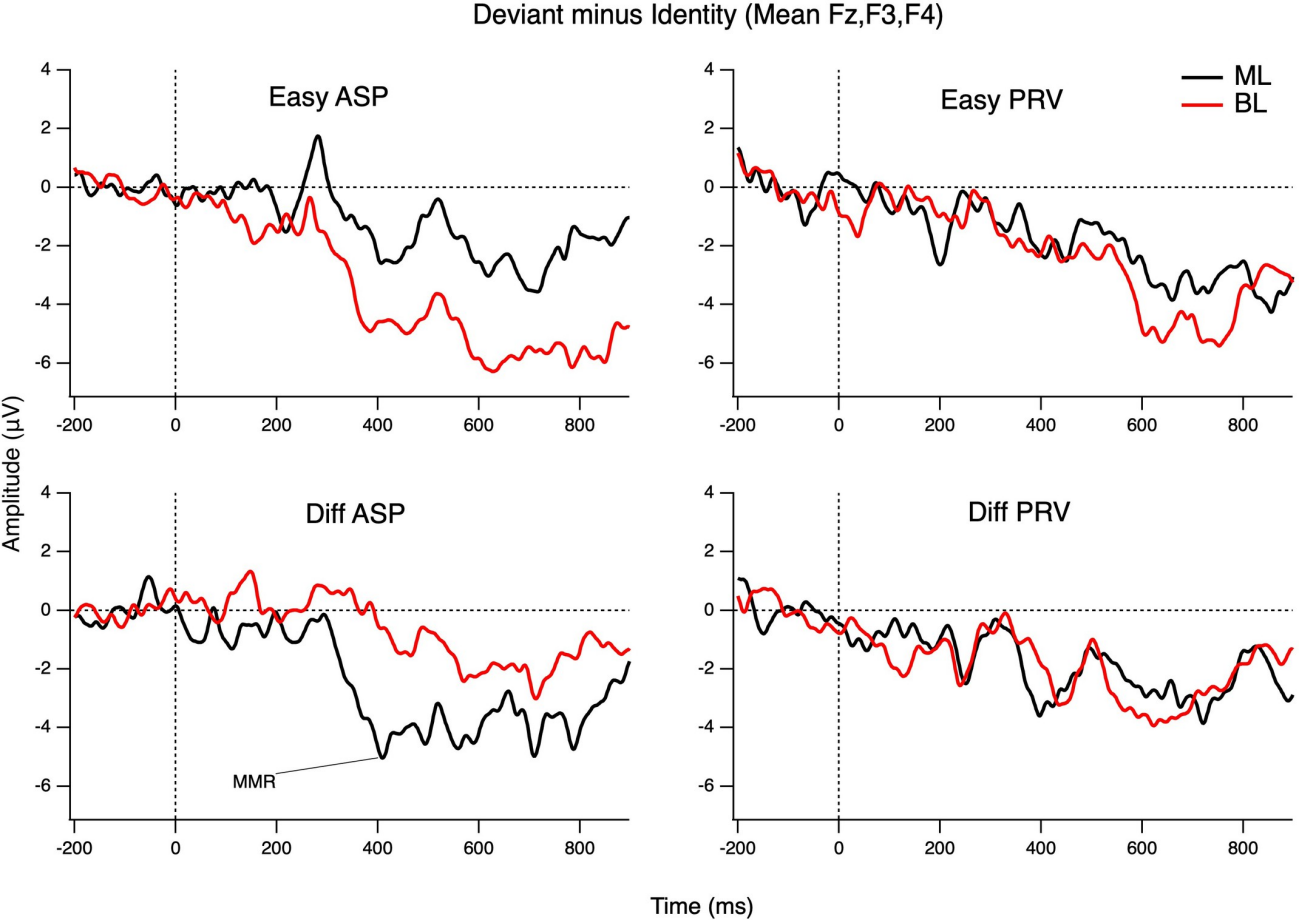
Participants’ iMMRs. Monolinguals’ (ML) vs. bilinguals’ (BL) iMMRs for each deviant averaged across frontal sites (Fz, F3, and F4). German-like Long-Lag (i.e., aspirated; ASP) plotted on the left; Italian-like Voicing Lead (i.e., prevoiced, PRV) plotted in the right.

As the difference between the ERPs to the deviants and their respective identities was only significant in the later time window (360–520 ms after stimulus onset), the iMMR within that time range was explored further and compared between the two groups (see Fig 3 for monolinguals’ vs. bilinguals’ iMMRs). A three-way mixed ANOVA with language (German-like VOT vs. Italian-like VOT) and difficulty level (Easy vs. Difficult contrast) as the within-subject variables and group (monolinguals vs. bilinguals) as the between-subject variable revealed a significant interaction for language x difficulty level x group *F*(1,30) = 4.637, *p* = .039, η_p_^2^ = .134 (see S2 Table in S1 File for children’s mean amplitudes and *SE* values for the late iMMR).

When addressing the question concerning how much language input is necessary for bilingual children to show processing that is similar to their monolingual peers, Pearson *r* correlation analyses yielded no significant relationships between bilingual children’s language experience (i.e., their relative amount of Italian vs. German language input) and any of their mean late iMMR amplitudes (*r*_*s*_ ranging from -.257 to .128, *p*_*s*_ > .261).

According to Cattani and colleagues, bilingual Spanish-English toddlers who received 60% or more of their language input in English matched their monolingual English peers with regards to their English language skills [47]. Thus, in the current study, a sub-selection of children in the group of bilinguals was made and children were grouped into a high German (i.e., low Italian) versus low German (i.e., high Italian) experience cohort, as quantified with the LBQ (high German = more than 60% German input, *n* = 10 in the difficult condition, *n* = 8 in the easy condition; low German = less than 40% German input, *n* = 3 in the difficult condition, *n* = 4 in the easy condition; bilinguals with more balanced language input were not included in this selection) to compare their iMMRs to the different VOT deviants to those of their monolingual German peers (*n* = 15 in the Difficult condition, *n* = 16 in the Easy condition).

Examination of children’s mean iMMR amplitudes averaged across Fz, F3 and F4 within the time window of 360–520 ms (see Table 5 for an overview) further shows that depending on the target language and the magnitude of the acoustic difference between the standard and the deviant, monolingual German versus bilingual high German/low Italian input versus bilingual low German/high Italian input children processed the different VOT stimuli differently. Of the three groups, monolingual German children showed the most negative MMR to the Difficult Long-Lag 36 ms VOT deviant. The high German/low Italian input showed an MMN, but less so than the German monolingual group. The low German/ high Italian input children showed the least negativity and even showed a positive peak within the target time window. The monolingual German and high German/low Italian input children showed a similar amplitude MMR to the Difficult Voicing Lead -36 ms VOT deviant, whereas the low German/high Italian input children showed the most negative MMR. Note that due to the small group sizes, no statistical analyses were conducted. However, further examination of children’s individual mean amplitude brain responses in the time range of interest to the different deviants (i.e., late iMMR 360–520 ms post stimulus onset) showed that in the monolingual subgroup, 32.25% (*n* = 5) of the participants showed a positive iMMR to the Easy Voicing Lead -112 ms VOT deviant (mean amplitude ranging from -9.85 μV to 6.99 μV), 25.0% (*n* = 4) showed a positive iMMR to the Easy Long-Lag 92 ms VOT deviant (mean amplitude ranging from -12.36 μV to 2.64 μV), 42.86% (*n* = 6) showed a positive iMMR to the Difficult Voicing Lead -36 ms VOT deviant (mean amplitude ranging from -12.17 μV to 6.31 μV), and 12.0% (*n* = 3) showed a positive iMMR to the Difficult Long-Lag 36 ms VOT deviant (mean amplitude ranging from -9.72 μV to 4.36 μV). In the subgroup of high German/low Italian input bilinguals, 14.29% (*n* = 1) of the participants showed a positive iMMR to the Easy Voicing Lead -112 ms VOT deviant (mean amplitude ranging from -8.41 μV to .28 μV), 14.29% (*n* = 1) showed a positive iMMR to the Easy Long-Lag 92 ms VOT deviant (mean amplitude ranging from -13.48 μV to 1.81 μV), 33.33% (*n* = 3) showed a positive iMMR to the Difficult Voicing Lead -36 ms VOT deviant (mean amplitude ranging from -3.73 μV to 5.59 μV), and 33.3% (*n* = 3) showed a positive iMMR to the Difficult Long-Lag 36 ms VOT deviant (mean amplitude ranging from -6.39 μV to 2.15 μV). Finally, in the subgroup of low German/high Italian input bilinguals, 50.0% (*n* = 2) of the participants showed a positive iMMR to the Easy Voicing Lead -112 ms VOT deviant (mean amplitude ranging from -7.92 μV to 4.98 μV), 25.0% (*n* = 1) showed a positive iMMR to the Easy Long-Lag 92 ms VOT deviant (mean amplitude ranging from 12.56 μV to 3.93 μV), none of the children showed a positive iMMR to the Difficult Voicing Lead -36 ms VOT deviant (mean amplitude ranging from -5.62 μV to -2.06 μV), and 33.33% (*n* = 1) showed a positive iMMR to the Difficult Long-Lag 36 ms VOT deviant (mean amplitude ranging from -4.53 μV to 7.38 μV).

**Table 5:** Overview of children’s mean late iMMR amplitudes averaged across Fz, F3, and F4 according to their language input situation.

		Bilinguals low German/ high Italian input	Bilinguals high German/ low Italian input	Monolinguals
German-like Long-Lag	iMMR “easy” (92 ms VOT)	*M* = -5.19, *SD* = 6.82	*M* = -3.56, *SD* = 4.46	*M* = -3.37, *SD* = 1.71
	iMMR “difficult” (36 ms VOT)	*M* = -.54, *SD* = 9.86	*M* = -1.73, *SD* = 2.96	*M* = -4.11, *SD* = 3.75
Italian-like Voicing Lead	iMMR “easy” (-112 ms VOT)	*M* = -.26, *SD* = 5.53	*M* = -3.12, *SD* = 3.08	*M* = -1.72, *SD* = 3.96
	iMMR “difficult” (-36 ms VOT)	*M* = -3.86, *SD* = 1.78	*M* = -1.25, *SD* = 3.99	*M* = -1.39, *SD* = 5.14

## 7 Discussion

This study explored the relationship between bilingual children’s language experience and the development of automaticity in neural speech sound discrimination (as indexed by the iMMR) and the influence of the degree of stimulus difference. It has been previously suggested that automaticity of processes supporting speech perception in (monolingual) children is not robustly established for fine-grained phonetic contrasts until four years of age [24]. More specifically, it has been proposed that neural speech sound discrimination is not initially automatic because it takes time and experience to establish robust, SPRs even for native-language phonological categories [14]. During a passive listening task, this study looked at four-to-five-year-old monolingual German and bilingual Italian-German children’s brain responses when processing natural German- versus Italian-like VOT stimuli that differed in their magnitude of acoustic difference from the standard. The results confirmed our hypotheses. Specifically, bilingual children differed from their monolingual peers with respect to the iMMR amplitudes, supporting language-experience dependent effects in (native) speech sound processing (RQ1, Hypothesis 1). Generally, as a group, bilinguals showed a tendency towards more negative ERPs compared to their monolingual peers. This finding may indicate increased (in)voluntary attention to the acoustic signal (RQ1, Hypothesis 2; cf. [40]). Furthermore, bilingual children with high Italian input who fell below a threshold of 40% of current German input did show immature signs (i.e., a more positive MMR) of processing the difficult German-like Long-Lag 36 ms VOT contrast (RQ2, Hypothesis 3).

### 7.1 Early versus late iMMR

Children’s ERPs were examined in an early time window (120–280 ms; early MMR) and a late time window (360–520 ms; late iMMR) [40]. While there were no significant differences in amplitude between children’s brain responses to the deviants and their respective identities in the early time window, there was robust evidence of neural speech sound discrimination for all deviant stimuli in the late time window. Although the nature of the functional mechanisms indexed by the late MMR (also called the LDN) are not yet fully understood, Yu and colleagues suggest that this late response may have the potential to evaluate the development of speech processing in toddlers, especially since “the MMR in the earlier time frame (150–400 ms) is often not significant to the subtle speech contrasts that are of particular interest in studies of language development” [40]. The results of this current study clearly support this claim. Alternatively, as suggested by Morr and colleagues, the early negativity may have been overlapped, and thus been masked, by a larger magnitude positive response, suggesting that at this age, children’s processing was still immature [23].

### 7.2 Development of automaticity as indexed by the MMR

Results indicate that Italian-German bilingual four- and five-year-old children and age-matched German monolingual controls show similar responses when processing long-lag and voicing lead VOT, except for the Difficult Long Lag 36 ms VOT stimulus. This is in line with the proposal of Crick and Koch [53], who suggested that highly salient differences can be processed (i.e., neurally discriminated) with fewer attentional resources than less salient distinctions. The authors further pointed out that less salient information can be made more apparent through the process of over-learning. The lack of a robust MMN to the Difficult Long-Lag 36 ms VOT stimulus in bilinguals suggests that over-learning of this subtle difference has not yet been achieved by bilinguals at four years of age. That is, bilinguals may not have yet accumulated sufficient experience with the German language to become fully automatic in processing German-like VOT [42].

The lack of a bilingual advantage in processing the Italian-like Voicing Lead -36 ms VOT and -112 ms VOT stimuli was unexpected. One interpretation may be that the bilingual participants were not yet automatic in discriminating the Italian VOT contrasts, considering that sufficient experience (in terms of time and amount of input) is necessary to establish automaticity of SPRs for phonological categories [14]. This account leads to the hypothesis that, when processing speech sounds at an attention-independent level, child bilinguals show automaticity earlier in their dominant language (i.e., the language more prevalent in children’s language input—German in the current study) compared to their non-dominant language [54,55]. An alternative interpretation may be that the Italian-German children were, in fact, automatic at processing, but that the German linguistic context during the experiment primed them to process according to the German categories [56–58]. However, Winkler and colleagues found no effect of a Finnish versus Hungarian linguistic context on their adult Finnish and Hungarian participants, contradicting this explanation [59]. Nevertheless, in this relatively young age group, linguistic context might be more important as children are still in the process of acquiring their two languages. Finally, one possible explanation for why monolingual German children showed neural signs of pre-attentive speech sound discrimination to Italian-like voicing lead is that they may in fact have come across instances of voicing lead in their everyday German language input. Although German voiced plosives are usually produced in the short-lag region, voicing lead has been found to be possible in the speech of German adults [60].

A pMMR [22] was only apparent in the subgroup data. One explanation for the absence of a pMMR could be that the distinction was easy enough for the MMN to dominate for all but the bilingual low-German-input children in the Difficult Long Lag 36 ms VOT condition. Alternatively, the pMMR may only be present in even younger children [23]. Finally, it is possible that the pMMR is more prominent at F3 than at Fz and F4 [24]. Further examinations should thus focus on the brain response’s topography.

### 7.3 Bilingual experience and the late iMMR

The late iMMR observed in this study may be equivalent to the LDN reported in previous studies [35–39]. Language experience did affect the late iMMR at a group level, but not at the individual level where none of the measures of language experience were significantly correlated with children’s late iMMR amplitudes. This finding suggests that other factors may play a role in the development of automaticity for speech sound processing (e.g., maturation effects [23], or attention, [37]). Nevertheless, when dividing the bilingual children into two subgroups of high German/low Italian input versus low German/high Italian input it was found that the group of bilinguals with relatively high rates of German input (> 60%) and the group of monolingual German children had a similar iMMR amplitude to the Voicing Lead -36 ms VOT deviant, whereas children with higher rates of Italian input (> 60%) showed a more negative response. Conversely, the same group of high Italian input (i.e., low German input) children responded with a positive peak to the German-like Long-Lag 36 ms VOT stimulus within the late iMMR time window, and even high German input children did not show a response as negative as their monolingual German peers.

The late time-frame of the MMN may reflect the subtle nature of the VOT differences. Shafer and colleagues suggested that with increasing age, the MMN moves earlier in latency, increases in amplitude and overlaps with, and thus reduces the amplitude of the pMMR [24]. The four-to-five-year-old bilingual Italian-German children may have been less automatic in processing the “difficult” German-like VOT contrast compared to their age-matched monolingual German peers. The difference between monolinguals and bilinguals for the Italian-like voicing lead was smaller, although the group of high Italian (i.e., low German) input children showed a more negative response to the Voicing Lead -36 ms VOT deviant, than the low input Italian (i.e., high German) and monolingual German children. This finding may indicate that the Italian-German bilinguals are not yet automatic in processing the Italian VOT cues, and thus, do not yet show a clear advantage over the monolingual German children. As suggested above, the overall increased negativity of the ERP responses for the bilingual group compared to monolinguals might indicate that they are covertly allocating attention to the stimuli [37]. Studies with adults have revealed that attention to an L2 contrast can increase MMN amplitude [30]. It will be important in future studies to directly manipulate attention to test whether the late iMMR is influenced by attention and to further examine whether the greater negative shift of the ERP observed for bilinguals compared to monolinguals disappears when attention is strictly controlled.

## 8 Limitations and future directions

One of the main limitations of this study was that due to Covid-19 contact restrictions and thus a repeatedly interrupted data collection process, sample sizes especially for sub-groups (i.e., high vs. low input distinction) in the bilingual sample were relatively small which prevented the application of several statistical analyses. Thus, some of the observations made, must be interpreted with caution as they rely solely on visual inspection of the data.

A direct measure of VOT input in German and Italian obtained through recordings of caregivers’ speech samples would have been informative concerning the model caregivers provide for their children in both languages. Similarly, an indicator of children’s individual perceptual boundaries in each language would have been beneficial to interpreting and explaining group differences.

Taken together, in addition to measures of attention-independent speech sound perception (e.g., using EEG), future studies should collect data on the quality of the target speech sounds in children’s language input as well as a behavioral equivalent to measuring speech sound perception to estimate their individual perceptual boundaries, and to obtain a complete picture of the complex interplay between language experience and speech sound processing.

## 9 Conclusion

This study replicated previous findings of automaticity in native speech sound perception in monolingual children by the age of four [24]. Children with bilingual experience, however, showed a different developmental trajectory. Even the group of high, German input bilinguals differed from age-matched monolingual German children when processing the subtle German-like VOT contrast. We predict that with increasing age and greater cumulative German experience, bilingual children’s brain responses will match that of their monolingual peers, as has been shown for 8-to-10-year-old bilingual children [39]. Even so, variability in perception and neural processing may persist and be related to language input quantity. For example, the early Spanish-English bilinguals in Hisagi and colleagues showed less robust neural discrimination of English vowels, and for some of these listeners, it is possible that differences in quantity or quality (i.e., a difference in boundary location) of English input could underlie the finding [34]. Only longitudinal data can reveal whether bilingual children will show similar patterns to the monolingual children at an older age or continue to show differences in neural discrimination, indicative of a difference in automaticity and/or of the boundary location for this phoneme contrast.

## Supporting information

S1 File S1 Table. Bilingual children’s individual language experience. Age of Onset (AoO) and relative amount of current Italian versus German experience (input and output). S2 Table. Early iMMR ERP and iMMR amplitudes. Overview of participants’ mean ERP Amplitude in μV to the different deviants vs. their respective identities and the early iMMR averaged across electrode sites Fz, F3, and F4 within the time window of 120–280 ms after stimulus onset according to group (monolingual German vs. bilingual Italian-German). S3 Table. Late iMMR ERP and iMMR amplitudes. Overview of participants’ mean ERP Amplitude in μV to the different deviants vs. their respective identities and the late iMMR averaged across electrode sites Fz, F3, and F4 within the time window of 360–520 ms after stimulus onset according to group (monolingual German vs. bilingual Italian-German).
(DOCX)

S2 File (XLSX)
