# Supplementary material for: Developing automaticity in neural speech discrimination in typically developing bilingual Italian-German and monolingual German children
Source: PLoS One. 2024 Oct 23;19(10):e0311820. doi: 10.1371/journal.pone.0311820 (PMC11498714; doi:10.1371/journal.pone.0311820)
Supplement: S1 File — S1 Table. Bilingual children’s individual language experience. Age of Onset (AoO) and relative amount of current Italian versus German experience (input and output). S2 Table. Early iMMR ERP and iMMR amplitudes. Overview of participants’ mean ERP Amplitude in μV to the different deviants vs. their respective identities and the early iMMR averaged across electrode sites Fz, F3, and F4 within the time window of 120–280 ms after stimulus onset according to group (monolingual German vs. bilingual Italian-German). S3 Table. Late iMMR ERP and iMMR amplitudes. Overview of participants’ mean ERP Amplitude in μV to the different deviants vs. their respective identities and the late iMMR averaged across electrode sites Fz, F3, and F4 within the time window of 360–520 ms after stimulus onset according to group (monolingual German vs. bilingual Italian-German). (DOCX) [file pone.0311820.s001.docx]

Supplementary Material

| **S1 Table. Bilingual children’s individual language experience**. Age of Onset (AoO) and relative amount of current Italian versus German input and output. | **Italian output** | 77% | 37% | 49% | 40% | 49% | 33% | 69% | 27% | 27% | 84% | 54% | 48% | 59% | 64% | 8% | 0% | 5% | 3% | 5% | missing data | 51% | 7% | 23% | 30% |
| --- | --- | --- | --- | --- | --- | --- | --- | --- | --- | --- | --- | --- | --- | --- | --- | --- | --- | --- | --- | --- | --- | --- | --- | --- | --- |
|  | **German output** | 23% | 63% | 51% | 60% | 51% | 67% | 31% | 73% | 73% | 16% | 46% | 52% | 41% | 36% | 92% | 100% | 95% | 97% | 95% |  | 49,00% | 93,00% | 77,00% | 70,00% |
|  | **Italian input** | 72% | 42% | 49% | 47% | 49% | 31% | 69% | 50% | 50% | 73% | 54% | 59% | 53% | 64% | 13% | 9% | 23% | 21% | 23% |  | 51% | 32% | 37% | 35% |
|  | **German input** | 28% | 58% | 51% | 53% | 51% | 69% | 31% | 50% | 50% | 27% | 46% | 41% | 47% | 36% | 87% | 91% | 77% | 79% | 77% |  | 49% | 68% | 63% | 65% |
|  | **AoO German** | 2.5 | 0 | 0 | 0 | 0 | 0 | 0 | 1 | 1 | 0 | 0 | 0 | 0 | 3 | 0 | 0 | 0 | 0 | 0 |  | 0 | 0 | 0 | 0 |
|  | **AoO Italian** | 0 | 0 | 0 | 0 | 0 | 0 | 0 | 0 | 0 | 0 | 0 | 0 | 0 | 0 | 0 | 0 | 0 | 2 | 0 |  | 0 | 0 | 0 | 0 |
|  | **Age (in months)** | 49 | 54 | 71 | 71 | 71 | 71 | 68 | 49 | 49 | 60 | 52 | 49 | 58 | 66 | 49 | 62 | 53 | 62 | 53 | 64 | 64 | 73 | 47 | 55 |
|  | **participant** | 204942 | 205492 | 207112 | 207152 | 207122 | 2071102 | 206862 | 2046142 | 2046132 | 106012 | 105222 | 104832 | 105862 | 306612 | 404912 | 406222 | 405352 | 406272 | 405362 | 406242 | 106492 | 407332 | 104572 | 105582 |

**S2 Table. Early iMMR ERP and iMMR amplitudes.** Overview of participants’ mean ERP Amplitude in µV to the different deviants vs. their respective identities and the early iMMR averaged across electrode sites Fz, F3, and F4 within the time window of 120-280 ms after stimulus onset according to group (monolingual German vs. bilingual Italian-German).

|  | | | Deviant | Identity | early iMMR |
| --- | --- | --- | --- | --- | --- |
| German-like Long Lag | Easy  (92 ms VOT) | Monolinguals  (*n* = 15) | *M* = 3.73,  *SD* = 3.94 | *M* = 4.90,  *SD* = 3.63 | *M* = -1.17,  *SD* = 3.99 |
|  |  | Bilinguals  (*n* = 22) | *M* = 2.46,  *SD* = 3.69 | *M* = 3.74,  *SD* = 3.89 | *M* = -1.28,  *SD* = 3.25 |
|  | Difficult  (36 ms VOT) | Monolinguals  (*n* = 15) | *M* = 7.46,  *SD* = 3.16 | *M* = 8.09,  *SD* = 3.76 | *M* = -.63,  *SD* = 3.19 |
|  |  | Bilinguals  (*n* = 22) | *M* = 5.63,  *SD* = 2.96 | *M* = 5.33,  *SD* = 3.44 | *M* = .30,  *SD* = 2.51 |
| Italian-like Voicing Lead | Easy  (-112 ms VOT) | Monolinguals  (*n* = 16) | *M* = 5.99,  *SD* = 4.08 | *M* = 7.02,  *SD* = 4.06 | *M* = -1.03,  *SD* = 3.42 |
|  |  | Bilinguals  (*n* = 22) | *M* = 4.74,  *SD* = 3.75 | *M* = 5.38,  *SD* = 3.32 | *M* = -.63,  *SD* = 3.87 |
|  | Difficult  (-36 ms VOT) | Monolinguals  (*n* = 14) | *M* = 7.84,  *SD* = 2.68 | *M* = 8.17,  *SD* = 2.67 | *M* = -.33,  *SD* = 3.21 |
|  |  | Bilinguals  (*n* = 21) | *M* = 4.40,  *SD* = 3.10 | *M* = 6.00,  *SD* = 2.99 | *M* = -1.59,  *SD* = 2.59 |

**S3 Table. Late iMMR ERP and iMMR amplitudes.** Overview of participants’ mean ERP Amplitude in µV to the different deviants vs. their respective identities and the late iMMR averaged across electrode sites Fz, F3, and F4 within the time window of 360-520 ms after stimulus onset according to group (monolingual German vs. bilingual Italian-German).

|  | | | Deviant | Identity | late iMMR |
| --- | --- | --- | --- | --- | --- |
| German-like Long Lag | Easy  (92 ms VOT) | Monolinguals  (*n* = 15) | *M* = -10.62,  *SD* = 5.30 | *M* = -7.26,  *SD* = 6.13 | *M* = -3.37,  *SD* = 4.71 |
|  |  | Bilinguals  (*n* = 22) | *M* = -12.55,  *SD* = 6.21 | *M* = -7.99,  *SD* = 6.73 | *M* = -4.57,  *SD* = 4.76 |
|  | Difficult  (36 ms VOT) | Monolinguals  (*n* = 15) | *M* = -10.34,  *SD* = 4.60 | *M* = -6.22,  *SD* = 5.25 | *M* = -4.11,  *SD* = 3.75 |
|  |  | Bilinguals  (*n* = 22) | *M* = -8.33,  *SD* = 6.44 | *M* = -7.59,  *SD* = 5.31 | *M* = -.74,  *SD* = 3.84 |
| Italian-like Voicing Lead | Easy  (-112 ms VOT) | Monolinguals  (*n* = 16) | *M* = -9.96,  *SD* = 4.37 | *M* = -8.24,  *SD* = 5.30 | *M* = -1.72,  *SD* = 3.96 |
|  |  | Bilinguals  (*n* = 22) | *M* = -10.49,  *SD* = 6.43 | *M* = -8.31,  *SD* = 5.57 | *M* = -2.18.,  *SD* = 3.94 |
|  | Difficult  (-36 ms VOT) | Monolinguals  (*n* = 14) | *M* = -6.41,  *SD* = 3.91 | *M* = -5.02,  *SD* = 3.59 | *M* = -1.39,  *SD* = 5.14 |
|  |  | Bilinguals  (*n* = 21) | *M* = -7.96,  *SD* = 5.37 | *M* = -6.15,  *SD* = 5.31 | *M* = -1.81,  *SD* = 3.97 |

# Details of the calculation of the percentage of children’s current Italian vs. German experience in a typical week of their life.

First, the number waking hours per week (h_awake_) was calculated based on an average estimate of a mean sleep duration per night 10.47 hours in preschool children (Scharf, Demmer, Silver, & Stein, 2013).

$$h_{awake}=\frac{total number of hours}{week}-\frac{mean sleep duration}{night}*7$$

Second, for every activity outside of the family home, the average time spent on this activity per week was calculated: (1) hours per week spent in kindergarten (h_kindergarten_), (2) hours per week spent with another caretaker outside of the core-family context (h_caretaker_), (3) hours per week spent with leisure activities (h_leisureact_), and (4) hours per week spent with friends (h_friends_).

$$h_{kindergarten}=\frac{hours in kindergarten}{day}*\frac{days in kindergarten}{week}$$

$$h_{caretaker}=\frac{hours with caretaker}{day}*\frac{days with caretaker}{week}$$

$$h_{leisureAct}=\frac{hours of leiusure activities}{day}*\frac{days with leisure activitiey}{week}$$

$$h_{friends}=\frac{hours with friends}{day}*\frac{days with friends}{week}$$

Third, the time children spent awake with their families (h_family_) was calculated by subtracting the time they spent outside of the family home from the number of waking hours.

$$h_{family}=h_{awake}-\left( h_{kindergarten}+h_{caretaker}+h_{leisureAct}+h_{friends} \right)$$

Next, we computed an estimate of the number of children’s Italian-hearing hours per week (i.e., their Italian input^[[1]](#footnote-1)^) by multiplying the weekly hours spent in the different contexts with percentage scale information provided by the parents for each respective activity (i.e., 100%, 90%, 50%, 25%, 10%, or 0%). Below, we will demonstrate this step for the calculation of the number of hours per week that children hear Italian within the kindergarten context ([h]Input_ItalianKindergarten_). For example, if a child spends 10 hours per week in the kindergarten where the language, they hear is 75% Italian, these 10 hours will be converted into 7.5 hours of Italian (and 2.5 hours of German respectively).

$$\left[ h \right]Input_{ItalianKindergarten}=h_{kindergarten}*[\%] Italian child hears in kindergarten$$

The same procedure was applied for Italian input from other caretakers ([h]Input_ItalianCaretaker_), during leisure activities ([h]Input_ItalianLeisureAct_) and during time spent with friends [h]Input_ItalianFriends_). Further, when calculating the number of hours children hear Italian within the family context [h]Input_ItalianFamily_), unlike Cattani and colleagues (2014) we did not assign weight to the input provided by different family members. Rather, we calculated a mean family Italian input score by averaging across the individual data provided for each family member living in the same household as the child.

$$\left[ h \right]\mathrm{Input}_{\mathrm{ItalianFamily}}=h_{\mathrm{family}}*\bar{X}\left( \%Italian_{mother}+\%Italian_{father}+\%Italian_{siblings}+\%Italian_{otheradults} \right)$$

Finally, the total number of hours of Italian input per week was calculated by adding up all of the Italian input measures; that sum was then divided by children’s average waking hours (h_awake_) in order to obtain the relative amount of Italian input.

$$\left[ h \right]Italian_{input}=\left[ h \right]Input_{ItalianFamily}+\left[ h \right]Input_{ItalianKindergarten}+\left[ h \right]Input_{ItalianCaretaker}+\left[ h \right]Input_{ItalianLeisureAct}+\left[ h \right]Input_{ItalianFriends}$$

$$\left[ \% \right]Italian_{input}=\frac{\left[ h \right]Italian_{input}}{h_{awake}}$$

The relative amount of German input could then easily be derived on the basis of the relative Italian input by subtracting it from 100%.

1. Note that the exact same approach described next was followed to obtain the number of hours children spoke Italian during a typical week of their everyday lives (i.e., their relative Italian output) [↑](#footnote-ref-1)
